# Nutrition as Prevention Factor of Gestational Diabetes Mellitus: A Narrative Review

**DOI:** 10.3390/nu13113787

**Published:** 2021-10-26

**Authors:** Radzisław Mierzyński, Elżbieta Poniedziałek-Czajkowska, Maciej Sotowski, Magdalena Szydełko-Gorzkowicz

**Affiliations:** Chair and Department of Obstetrics and Perinatology, Medical University of Lublin, 20-954 Lublin, Poland; elzbietapc@yahoo.com (E.P.-C.); maciek13011@wp.pl (M.S.); mszydelko@interia.pl (M.S.-G.)

**Keywords:** gestational diabetes mellitus, diet and dietary supplements, primary prevention, nutrition

## Abstract

Gestational diabetes mellitus (GDM) is defined as a glucose tolerance disorder with onset or first recognition during pregnancy. GDM is associated with several adverse maternal and neonatal outcomes. Management to reduce the incidence of GDM could decrease the incidence of these complications. Modification of nutrition in the prevention of GDM is postulated. The vital issue in GDM prevention is the implementation of proper dietary patterns, appropriate physical activity, and a combination of diet and lifestyle modifications. However, intervention studies examining the effects of diet and lifestyle on GDM prevention are contradictory. The aim of this study was to review the scientific evidence on nutritional prevention strategies, including diet and supplementation of some substances such as probiotics, micro/macroelements, fiber, myoinositol, and vitamins that may be effective in reducing the risk of GDM. The presented article is a narrative review. This article indicates that certain nutritional factors may have some benefit in preventing GDM. However, further studies in a variety of populations and large groups of patients are needed. At present, no definitive conclusions can be drawn as to the best intervention in the prevention of GDM.

## 1. Introduction

Gestational diabetes mellitus (GDM) is defined as a glucose tolerance disorder with onset or first recognition during pregnancy [1]. GDM is the most common metabolic disorder of pregnancy that occurs in 5 to 25% of all pregnancies, depending on the ethnicity and population studied, the screening method used, and the diagnostic tests employed [2]. In 2019, 20.4 million of 129.5 million live births were affected by hyperglycemia in pregnancy, and 83.6% were affected by GDM, which shows the importance of this complication [3]. GDM is diagnosed if at least one of the threshold values are met: fasting glucose level of 5.1–6.9 mmol/L (92–125 mg/dL), at first hour ≥10.0 mmol/L (180 mg/dL) and at second hour 8.5–11.0 mmol/L (153–199 mg/dL) [4].

The importance of this problem and preventing GDM is emphasized by the increased possibility of either maternal or fetal complications in pregnancies with GDM compared to non-complicated pregnancies [5]. Pregnant women and their unborn children have a higher risk of adverse outcomes (miscarriage, preterm birth, preeclampsia, cesarean section, macrosomia to shoulder dystocia and trauma during delivery, asymmetrical intrauterine growth retardation (aIUGR), stillbirth, neonatal hyperbilirubinemia, hypoglycemia, hypocalcemia and polycythemia, respiratory distress syndrome, and hypertrophic cardiomyopathy) [6,7]. It has been proven that GDM is also connected with antenatal depression more so than in normal pregnancy [5]. Women with GDM are also at higher risk for the development of GDM in subsequent pregnancies, type 2 diabetes mellitus (T2DM), and cardiovascular morbidity and mortality in future life [8,9]. Their offspring also have a significantly increased risk of developing fostering obesity, metabolic syndrome, and impaired glucose metabolism in later life [10].

An adaptation of maternal metabolism with higher nutritional requirements to improve fetal growth is observed [9]. Insulin secretion increases in the first trimester of pregnancy, but decreased insulin sensitivity is observed in the second and third trimester [11]. It is suggested that it is caused by placental production of hormones such as leptin, progesterone, cortisol, estrogen, placental growth hormone and lactogen, which increase insulin resistance, whereby women’s insulin secretion is insufficient [12]. It is believed that pre-pregnancy decreased maternal insulin sensitivity and pre-conception insulin resistance, impaired insulin response observed during pregnancy, and dysfunction of insulin-producing β-cells are the pathophysiological background of GDM development [11]. However, moderate insulin resistance and hyperinsulinemia are observed during physiological pregnancy, which also provides suitable sufficient nutrient supply for the metabolic needs of the rapidly growing fetus.

## 2. Risk Factors for GDM

Epidemiological studies have identified several risk factors associated with developing GDM. Most of those risk factors are strictly connected with insulin sensitivity or β-cell of pancreas function to synthesize insulin [5].

Although many genetic and environmental factors may contribute to developing GDM, maternal obesity and nutritional insufficiencies are vital factors that make GDM one of pregnancy’s most important clinical complications [13]. It has been proven that one of the most substantial risk factors is obesity before pregnancy (defined as body mass index (BMI) > 30 kg/m^2^) [14]. Therefore, it is understandable that the prevalence of GDM in a given population is similar to that of T2DM. African, Hispanic, and some Asian female populations are more likely to develop GDM than Caucasian women [15,16].

Other risk factors include excess weight gain during pregnancy, advanced maternal age, and GDM in previous pregnancy [17]. The findings of the other authors also highlight the association between increased gestational weight gain and the risk of GDM [18].

It has been published that higher dietary fat and lower carbohydrate intakes during pregnancy appear to be associated with a higher risk for GDM independent of pre-pregnancy BMI [18].

It is suggested that some genetic factors may increase the risk of GDM. There are no typical genetic factors that could be responsible for GDM pathogenesis. It has been published that some variants in several essential genes are correlated with the pathogenesis of insulin resistance, including the receptors of prolactin and melatonin [19,20]. Abnormal production or secretion of adipokines in insulin resistance is also observed. The dysregulation of the metabolism of several adipokines may play an important role in the pathogenesis of GDM [21,22]

Polycystic ovary syndrome (PCOS), with metabolic and hormonal dysfunction, also increases the risk of GDM. However, it seems to be related to obesity and increased maternal age rather than to PCOS itself [23].

The lifestyle factors in early pregnancy, including nutritional factors, appear to be important in the pathogenesis of GDM [24]. It is suggested that most of these factors have a prolonged influence on patients, implying that lifestyle modifications should be introduced before pregnancy, not only during its duration [25].

Finally, the risk factors for developing GDM can be divided into modifiable and non-modifiable factors. The age, ethnicity, PCOS, GDM in previous pregnancies, and genetic factors such as a family history of diabetes can be classified as non-modifiable risk factors of GDM, and the modifiable ones are cigarette smoking by pregnant patients and/or their parents, pre-pregnancy BMI > 30 kg/m^2^, low physical activity before and during pregnancy, and many dietary factors [5].

## 3. Materials and Methods

A literature search in the electronic databases PubMed and MEDLINE was performed. We focused on dietary interventions in GDM prevention. A detailed analysis of eligible publications in the literature using MESH terms such as “gestational diabetes mellitus,” “dietary interventions,” and “GDM prevention” as keywords were conducted. Included studies had to research factors that may be used as supplementation or diet to prevent GDM with a sufficiently large observation group to draw conclusions that can be generalized. Only publications in English were considered. The references included in these selected publications were also taken into account to find additional relevant articles. We analyzed the following types of articles: population-based studies, reviews, systematic reviews, meta-analyses, and clinical trials. The presented article is a narrative review, and we did not perform a systematic review and could therefore not perform a meta-analysis.

## 4. Prevention of GDM

Interfering in patients’ lifestyle in diet and physical activity and glucose level control can help reduce complications of pregnancies observed in GDM patients. The essential factor is close monitoring of glucose levels. Fasting glucose levels should be maintained below 5 mmol/L (90 mg/dL) and 1 h after main meals below 7.8 mmol/L (140 mg/dL). Dietary control is the first line of treatment of GDM [26]. If nutritional control is not adequate, pharmacotherapy is introduced. In addition to necessary diet and pharmacotherapy, other primary actions are also needed.

Currently, there is no systematized strategy for GDM prevention. It is believed that due to the parallels in the pathogenesis and risk factors of GDM and T2DM, it is likely that agents that are effective in preventing T2DM may also be effective in preventing GDM. These factors include eating patterns, physical activity, a decline in obesity rates, and weight gain during pregnancy. It is important to identify population factors to reduce rising GDM rates, as is the case with T2DM [27].

Evidence shows that weight loss is not recommended during pregnancy (even in obese women), suggesting adequate weight and nutritional control before conception and between pregnancies. Therefore, in some groups of patients with diagnosed insulin resistance, such as PCOS or metabolic syndrome patients, in the prevention of pregnancy complications, including GDM, it is suggested to introduce metformin therapy. Glueck et al. observed a 10-fold decrease in the incidence of GDM in this group of patients [28]. However, daily administration of 3 g of metformin in non-diabetic obese pregnant patients from early pregnancy to delivery did not reduce the incidence of GDM [29].

It seems that the important issue in GDM prevention is the implementation of proper dietary patterns and appropriate physical activity, and a combination of diet and lifestyle modifications are considered. In recent years, a beneficial effect on insulin sensitivity of other factors, such as fiber, myoinositol, and probiotics, has also been observed.

A beneficial effect of micronutrients such as vitamin D, iron, and selenium on glucose metabolism has been noted.

**Figure 1:**
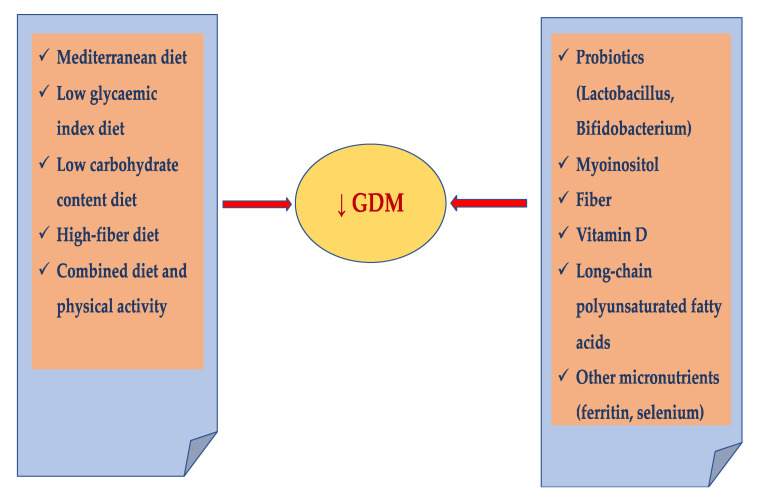
Diet and supplements as potential factors in GDM prevention.

The study aimed to review the scientific evidence on nutritional prevention strategies, including diet and supplementation of some substances such as probiotics, micro/macroelements, fiber, myoinositol, and vitamins that may be effective in reducing the risk of GDM (Figure 1).

### 4.1. Individualized Diet

It has been suggested that pregnancy provides an opportunity for healthcare professionals to modify lifestyle patterns towards acquiring healthier habits both for the individual patient and for society [30]. It has been proven that introducing a healthier eating pattern, such as the Mediterranean diet, and reducing consumption of iron-rich foods, sugar-sweetened cola, potatoes, fatty foods, and sweets may decrease the frequency of GDM, especially in high-risk populations and before pregnancy [31,32]. Combining of all these agents can also improve metabolism, counteract the formation of free radicals, and alleviate systemic oxidative stress [33].

Several diets have been analyzed to reduce the risk of GDM and prevent maternal and fetal complications. A low-glycemic-index diet, diets with energy restrictions (1/3 reduction of calories), low carbohydrate content, and high-fiber diets were discussed [34,35,36,37,38]. According to some recommendations, the currently available evidence on the prevention of GDM revealed that most interventions performed during pregnancy have a non-significant effect on the prevention of GDM [39]. However, many studies attempted to find an optimal diet that could be implemented during pregnancy planning or in early pregnancy that could reduce the risk of GDM.

It has been published that a diet with a low glycemic index reduces the increase in insulin resistance observed in pregnancy, thereby decreasing the risk of GDM [40]. However, in an overview of Cochranes Reviews, the benefit or harm of a low glycemic index diet versus a moderate–high glycemic index diet on the risk of GDM is unknown: RR 0.91, 95% CI 0.63 to 1.31 were noticed [41].

Diet-based modifications also revealed the most significant reduction in weight gain during pregnancy compared to other methods. Decreased weight gain during pregnancy may have contributed to the decline in the incidence of GDM [42].

There are some controversies regarding carbohydrate-restricted diets as prevention of GDM and as a dietary intervention in GDM patients. It has been suggested that replacing dietary carbohydrates with fat may have a detrimental effect on the mother’s insulin resistance and may lead to excessive fat accumulation in the fetus [43]. Therefore, this diet is probably more important for preventing macrosomia than for the prevention of GDM.

Although several studies suggested that restricted-energy diets and carbohydrate diets can minimize weight gain during pregnancy without increasing the risk of ketonuria and/or intrauterine growth restriction, one should be very careful in prescribing such diets to pregnant women [18]. The Institute of Medicine (IOM) recommends 46– 65 energy percent (E%) from carbohydrates and a minimum of 175 g of carbohydrates daily to ensure appropriate fetal growth and cerebral development and function [44,45].

Another nutrition pattern that seems to lower the risk of GDM appearance is the substitution of some energy from animal proteins for vegetable ones. According to Kapur et al., a low-carbohydrate pre-pregnancy diet rich in animal protein and fats is positively correlated with the risk of GDM, whereas in a low-carbohydrate pre-pregnancy diet rich in protein and fat from plant sources, this risk is not observed [46]. It is suggested that women of childbearing age on a low-carbohydrate diet can implement consuming vegetables over animal sources of protein and fat to decrease the risk of GDM [47,48,49]. The results of a cohort study of 21,457 patients suggest that substitution of only 5% energy gained from animal proteins for vegetable ones could reduce the risk of developing GDM by 51% (RR (95% CI), 0.49 (0.29–0.84)). On the other hand, changing the source of 5% of energy from carbohydrates to animal protein resulted in an increased risk of GDM by 29% (RR (95% CI), 1.29 (1.08–1.54); *p* = 0.006). Furthermore, a lower risk of GDM was also obtained by replacing one serving of red meat per day with healthy substitutes, like poultry, fish, legumes, or nuts, lowering the risk of GDM by 29% (RR (95% CI), 0.71 (0.54–0.94)), 33% (RR (95% CI), 0.67 (0.46–0.98)), 33% (RR (95% CI), 0.67 (0.51–0.88)), and 51% (RR (95% CI), 0.49 (0.36–0.66)), respectively [34]. The possible mechanisms of the effectiveness of the above replacement are the acceleration of the destruction of pancreas islets by high protein intake, which is correlated mostly with animal protein [50]. According to the above, even with the beneficial effects of proteins on energetic homeostasis such as inducing satiety or increased thermogenesis, increased animal protein intake results in a rise in GDM risk [50].

#### Mediterranean Diet

The Mediterranean diet (MedDiet) is widely recognized as one of the healthiest forms of nutrition. It is defined by plant-rich meals with olive oil as the main source of additional fat, with low consumption of processed meat (1 or fewer servings per week); red meat (less than two servings per week), moderate consumption of white meat (2 servings per week), eggs (2–4 servings per week), and fish or seafood (2 or more servings per week); and high consumption of low-fat dairy products (2 servings per day) [51]. It has been published that the MedDiet with additional pistachios and virgin olive oil can be used to prevent GDM [51,52]. In the study published by Asaaf-Balut et al., out of 874 women included in the study between 8 and 12 weeks of gestation with normal glycemia, 440 were assigned to the control group and 434 were assigned to the intervention group, which relied on the MedDiet with the addition of pistachios and virgin olive oil. The intervention was revealed to be effective with a 17.1% risk of developing GDM compared to 23.4% in the control group (*p* = 0.012). Additionally, in the intervention group, there were significantly lower levels of fasting blood glycemia and HbA1c at checkups at 24–28 and 36–38 weeks of gestation than in the control group. In the control group, when GDM was diagnosed, the women were more likely to require insulin therapy (32%) compared to the intervention group (19%) (*p* = 0.002). Several maternal and neonatal outcomes were also improved, including perinatal trauma, emergency cesarean section, and large and small gestational age (>90th and <10th percentile) [52]. However, the same authors published another study where intervention recognized only the MedDiet without additional ingredient enrichment and observed no difference in GDM prevalence in the study group compared to the previous study. This study also analyzed the influence of the MedDiet on women diagnosed with GDM during research. The authors concluded that intervention could reduce perinatal complications similar to women with normal glucose tolerance [53]. Those complications are probably associated with inflammatory processes and/or organism response to hyperglycemia and inadequate control of glycemia. It could be responsible for the effectiveness of the MedDiet, which has antioxidant and anti-inflammatory properties, and the potential to improve insulin sensitivity.

In a study conducted in the University Hospital of Granada, Spain, a protective effect of adherence to the MedDiet before the pregnancy for preventing GDM has been noticed [48].

In summary, in a meta-analysis published by Rogozińska et al., interventions that were mainly based on diet decreased the rates of GDM by 33% (RR 0.67; 95% CI 0.39, 1.15). There was a significant difference according to the BMI for diet-based intervention (*p* = 0.04). A statistically significant reduction in GDM risk was observed in the subgroup composed of obese and overweight women (RR 0.40; 95% CI 0.18, 0.86) [54]. There were no differences between the two groups for the mixed (diet and lifestyle) approach (RR 0.95; 95% CI 0.89, 1.22) [54]. However, the authors concluded that GDM does not appear to be prevented by dietary or mixed approach interventions. They suggested a trend towards a beneficial effect in patients of mainly diet-based interventions, with the potential to significantly reduce the risk of GDM only in obese and overweight women [54]. However, diet-based interventions seem to prevent GDM. This can be due to the following causes: personalized diet and ingredients, change in weight gain during pregnancy, and the effects of dietary supplements. There is no evidence that dietary interventions significantly reduced the rate of preterm deliveries. However, they reduced the cesarean section rate, the rate of the induction of labor, and the rate of gestational hypertension and preeclampsia [55,56].

### 4.2. Physical Activity

It has been published that dietary intervention can decrease the risk of developing GDM and the percentage of macrosomic newborns born in obese pregnant patients, but physical activity interventions did not have the same effect [57]. However, the American Dietetic Association and the American Nutrition Society stated that women who exercised during pregnancy gained significantly less fat and that moderate exercise may reduce the risk of GDM [58]. In two meta-analyses, a 28–31% reduction in the risk of GDM and a mean difference of about 1.1 kg in gestational weight gain between the study and control groups using structured, low- to moderate-intensity exercise programs that contained an aerobic component were observed [59,60].

Moreover, when the exercise program was carried out throughout pregnancy, the decrease in the risk of GDM seemed to be even greater (36%) [60]. However, Han et al., in their study, compared two groups: first, with supervised exercise sessions and exercise advice and second, with regular daily activities, and did not observe a significant difference in GDM risk between these groups [61]. When interpreting these observations, we have to remember limitations such as no assessment of physical activity outside the program, no standardized interventions, differences in study design and intervention content, an overall a not very considerable number of studies, and limited adherence to intervention protocols in some analyses [26].

### 4.3. Combined Diet and Physical Activity

It has been suggested that the best effect in the prevention of GDM can be achieved by combining exercise and diet. However, some studies showed no positive effect in patients with a mixed approach that combined diet and physical activity [42,54]. It seems that it may also be related to insufficient patient compliance with the recommendations regarding physical activity. The most extensive study based on both diet and modifications of lifestyle in pregnancy, the LIMIT study, found no benefit of interventions for GDM and other maternal outcomes, including weight gain during pregnancy [55]. However, in the study published by Thangaratinam, the combination of dietary recommendations and physical activity has been shown to significantly decrease the risk of preeclampsia by 26% and the risk of having a large-for-gestational-age (LGA) infant by 27% among healthy pregnant patients or those who are overweight or obese in comparison to non-obese patients receiving standard care [62]. In addition, in an overview of Cochrane Reviews, it has been noticed that a combined diet and exercise interventions during pregnancy versus standard care possibly reduced the risk of GDM: RR 0.85, 95% CI 0.71 to 1.01 [41]. However, in the other overview performed by Bain et al., no apparent difference in the risk of developing GDM for women receiving a combined diet and exercise intervention was noticed compared with women receiving no intervention [63].

The study that directly demonstrates the effectiveness of diabetes prevention included changing the whole lifestyle of the patients with an individualized diet and closely monitored physical activity. The authors observed 269 high-risk pregnancies, divided into an intervention group (144 pregnancies) and a control group (125 pregnancies). During the study, the control group received standard medical care, whereas the intervention group took part in group sessions with dietitians, with personal, individualized education meetings on diet, weight, and physical activity control by qualified staff. The examined intervention has reduced the risk of developing GDM in high-risk patients by 39%, with a probability of incidence of 13.9% in the intervention group and 21.6% in the control group ((95% CI 0.40–0.98%) *p* = 0.044) [36]. The high effectiveness of the intervention may be a result of including intervention early at 13.3 weeks of gestation (median) and the requirement of inclusion, which was high-risk pregnancy with a BMI of ≥30 kg/m^2^ or GDM in previous pregnancies. It should be noted that neonatal and other maternal outcomes such as hypertensive disorders, cesarean section rate, and fetal macrosomia were similar in both groups. The conclusions from this study are very promising and indicate that the prevalence of GDM can be decreased by lifestyle interventions; however, research on larger, more heterogenic groups is required.

### 4.4. Probiotics

Recently, the role of intestinal microbiota in regulating metabolism has become a hot topic of research. Thus, microbiota may play an essential role in the pathogenesis of obesity and may also significantly affect glucose homeostasis [64].

It has been published that the administration of probiotics containing Lactobacillus rhamnosus and Bifidobacterium lactis can decrease the frequency of GDM. Luoto et al. revealed that adding probiotics to the diet (Lactobacillus rhamnosus GG and Bifidobacterium lactis Bb12) can decrease the frequency of GDM. Patients without chronic metabolic diseases were included to research in the first trimester of pregnancy. Studied patients were divided into groups: a control group and intervention group, where patients were consulted by a nutritionist to implement a diet that complied with the recommendations. The intervention group was divided into a group receiving probiotics and a group receiving a placebo. The researchers observed the frequency of GDM: 13% (diet/probiotics) versus 36% (diet/placebo) and 34% (control), *p* = 0.003. According to the authors, this could be explained by the fact that consumption of probiotics may reduce the risk of GDM because these microorganisms can affect the intestinal microflora by modifying food polysaccharide fermentation and improving intestinal barrier function [65]. Luoto et al. also noted the ability of probiotics to modulate inflammatory pathways. There were no significant differences in pregnancy duration and outcomes, mean birth weight of the newborns, or 5th min. Apgar score and weight gain through the first 24 months of life between groups [64]. In a recently published study, Homayouni et al. also suggested that probiotic efficiency may be related to changes in the degradation of polysaccharides. The second postulated mechanism includes increasing intestinal permeability induced by naturalization of the intestinal biotome and secretion of pro-inflammatory mediators, which contribute to limiting local and systemic inflammation, resulting in the strengthening of the immune system [66].

In a meta-analysis performed by Rogozińska et al., the risk of GDM was reduced by 60% for probiotics (with diet) in comparison to standard care (RR 0.40; 95% CI 0.20, 0.78; *p* < 0.01) [54].

Callaway et al. performed a double-blind, randomized controlled trial (RCT) to analyze whether probiotics (Lactobacillus rhamnosus and Bifidobacterium animalis ssp lactis) given from the second trimester of pregnancy in overweight and obese patients may decrease the risk of GDM. This study did not confirm the effectiveness of such a strategy [67]. However, two meta-analyses revealed that the application of probiotics was correlated with an improvement in glucose and lipid metabolism in pregnant patients and could decrease the risk of GDM [61,68].

Lindsay et al. analyzed the use of probiotics in women with already diagnosed GDM. In an Irish RCT, 149 patients with GDM received either a probiotic capsule (Lactobacillus salivarius) or a placebo once a day from diagnosis of GDM to delivery and no effect on glycemic control was found [69].

### 4.5. Myoinositol

Myoinositol, an isomer of inositol, is one of the intracellular mediators of the insulin signal and is associated with insulin sensitivity in type 2 diabetes [70]. The best sources of inositol are grains, meat, fresh fruits and vegetables, corn, and legumes. The average dietary intake contains 1 g of inositol/day.

Matarelli et al. and D’Anna et al. reported that supplements containing myoinositol can reduce the incidence of GDM in pregnant patients (Matarrelli et al.: RR = 0.127; 95% CI, 0.032–0.502; *p* = 0.001 and D’Anna et al.: OR = 0.34; 95% CI, 0.17–0.68; *p* = 0.001) and seem to act as an insulin sensitizer [71,72].

Another analyzed report concerned the daily supplementation of 4g myoinositol with 400 µg of folic acid versus 400 µg of folic acid only. Additionally, both groups received identical diet prescriptions. The results revealed a decrease in the homeostasis model assessment of insulin resistance (HOMA-IR) in the intervention and control group, suggesting the relevance of diet, but additionally, in the intervention group, there was a significant increase in adiponectin concentration in plasma. The intervention group’s HOMA-IR decreased by 50%, and adiponectin concentration increased by 28% compared to 29% and 0% in the control group, respectively. The intervention also reduced fasting glucose by 16.4% compared to an irrelevant change in the control group [73].

As other studies show, myoinositol can also be used to prevent GDM for women with type 2 diabetes in their family history. One hundred ninety-seven patients were divided into the intervention group (99 women) treated with 2 g myoinositol and 200 µg folic acid twice a day, and the placebo group (98 women). Researchers achieved a significant reduction of GDM appearance to 6% in the intervention group and 15.3% in the placebo group (*p* = 0.04). Although no significant differences in the occurrence of gestational hypertension, caesarian section, preterm deliveries, or neonatal distress respiratory syndrome were observed, the intervention showed potential to reduce macrosomia, with 0 cases in the studied group compared to 7 cases of >4000 g newborns in the control group [74]. In the other studies, myoinositol supplements (4 g) decreased the incidence of GDM by 50–60% in high-risk pregnant women (overweight, obese, or with type 2 diabetes in first-degree relatives) [75,76]. It has also been noticed that myoinositol may decrease plasma glucose concentrations in insulin-resistant requirements such as PCOS and GDM in the third trimester of pregnancy [77].

In a meta-analysis performed by Rogozińska et al., the risk of GDM was reduced by 60% for myoinositol in comparison to standard care (RR 0.40; 95% CI 0.16, 0.99; *p* = 0.05) [54]. In an overview of Cochrane Reviews, it was noticed that myoinositol supplementation during pregnancy versus control possibly reduced the risk of GDM: RR 0.43, 95% CI 0.29 to 0.64 [41].

The mechanism of the beneficial effect of myoinositol on metabolic mechanisms is not fully understood. It may exert an intracellular effect directly on the activation of acetyl CoA carboxylase and induce lipogenesis stimulation. It has been proposed that the binding of insulin to specific receptors stimulates D-chiro-inositol, facilitating the transport to the inside of the cell [73]. This explains how myoinositol interacts in the insulin-signaling cascade. Another hypothesis suggests that myoinositol is a precursor to D-chiro-inositol, which contains inositol phosphoglycate in the extracellular matrix of cells. It has been postulated that insulin binding to specific receptors can stimulate D-chiro-inositol, improving transport to the inside of the cell. It clarifies the role of myoinositol in the insulin-signaling cascade [76].

### 4.6. Fiber

Higher intakes of fiber have been reported to be beneficially associated with glucose homoeostasis in observational studies. In a prospective study conducted in pregnant women, the authors tested a group of 13,110 women. As prevention factors, the authors analyzed the influence of fiber intake: its source and amount. The achieved result constituted a reduction of GDM diagnoses in the group with 10g/day fiber intake without distinguishing between the sources of its origin by 26%, in the group taking 5g/day cereal fiber by 23%, and in the group with 5g/day fruit fiber intake by 26%. The researchers did not notice any correlation between vegetable fiber intake and risk of GDM. Additionally, retrospective analysis of the impact of cereal fiber and dietary glycemic load showed that women with higher glycemic load and lower cereal fiber intake were up to 2.15 times more likely to develop GDM (95% CI 1.04–4.29 *p* = 0.02) [77]. Due to the study’s method, we can only analyze the effects of diets of observed patients, but there was no intervention group for which added fiber could be examined. In the recently published study performed by Zhang et al., women with the highest fiber intake before pregnancy, in the first trimester or the second trimester, had an approximately 11%, 17%, or 18% lower risk for GDM, respectively (*p* for trend ≤ 0.03) [78].

The main dietary fiber GDM risk reduction mechanisms could be diminishing appetite and lowering energy intake, resulting in reduced adiposity and HOMA-IR. Additional fiber may also extend the time of gastric food passage, reducing glucose absorption rapidity and hence insulin level in plasma [79].

### 4.7. Vitamin D

There is some evidence that an insufficient supply of vitamin D in early pregnancy may be correlated with an increased risk of GDM [80,81]. In the prospective cohort study performed by Bao et al., the authors analyzed vitamin D intake in supplementation and diet and its influence on GDM risk. The study resulted with the conclusion that supplementation of vitamin D 1—399 IU/day and ≥400 IU/day—reduced the risk of GDM by 20% (RR = 0.8; 95% CI 0.67–0.96) and 29% (RR = 0.71; 95% CI 0.56–0.9), respectively (*p* = 0.002). Surprisingly, dietary intake of vitamin D was also associated with the risk of GDM, but without statistical significance [82].

A meta-analysis of 20 studies researching the influence of vitamin D deficiency, including 16,515 patients, demonstrated that it could increase GDM risk by 45% (RR 1.45; 95% CI 1.15–1.83; *p* < 0.001). The analysis also emphasized cofactors that intensify the influence of vitamin D deficiency on GDM risk, such as age (>30; OR 1.47; 95% CI 1.12–1.92 *p* = 0.005) or patient origin (developed countries; OR 1.44 95% CI 1.09–1.90; *p* = 0.011) [78]. However, in the Cochrane review of vitamin D interventions including 15 studies, the benefits of using vitamin D in the prophylaxis of GDM were not demonstrated [83]. In the other systematic review and meta-analysis published by Perez et al., no difference for GDM was found [84].

The mechanism of action of reducing GDM risk by vitamin D is not fully understood. It can be an effect of multiple mechanisms, such as stimulation of insulin receptor expression or intensification of glucose transport mediated by insulin [85].

In the summary of the data on vitamin D use in the prevention of GDM, it should be stated that further research and observations are necessary to publish unambiguous recommendations.

### 4.8. Long-Chain Polyunsaturated Fatty Acids

Long-chain *n*-3 polyunsaturated fatty acids (LCPUFAs) are beneficial in potentiating the effects of insulin and enhancing glucose tolerance in both animals and humans [86]. However, the results of studies on the use of LCPUFAs in the prevention of GDM did not show clear benefits from their use [87]. In a meta-analysis of six randomized controlled trials of LCPUFA supplementation in healthy pregnant women published by Szajewska et al., no beneficial effects on the incidence of GDM or other pregnancy complications, except for statistically significant extension of the duration of pregnancy, were observed [88].

### 4.9. Other Micronutrients

The literature suggests that iron influences glucose metabolism. In a cohort study published by Bo et al., a correlation between the intake of iron supplements and abnormal results of an oral glucose tolerance test during pregnancy was found [89]. In a systematic review and meta-analysis of 15 observational studies published by Fu et al., a positive association between ferritin level and GDM was found (RR 1.53; 95% CI: 1.17–2.00) [90]. Because ferritin is an acute phase reactant, the increase in ferritin level may be caused by inflammation and not by increased iron stores in the body. Therefore, it is necessary to conduct further studies to confirm the relationship of iron concentration with the occurrence of GDM.

It has been suggested that selenium exhibits insulin-like properties, which can maintain normal glucose uptake, regulate cellular glucose utilization, and decrease the severity of insulin resistance. Some studies reported that patients with GDM showed lower levels of serum selenium than healthy pregnant patients [91]. However, no significant association between serum selenium and GDM was documented.

In a systematic review and meta-analysis of six studies comparing the selenium concentrations in healthy pregnant women and GDM women, the serum selenium levels were lower in women with GDM and women showing subclinical levels of glucose intolerance [92]. However, well-designed prospective studies are needed to understand the correlations between selenium status and GDM risk.

## 5. Conclusions and Clinical Implications

Due to the risk for the mother and fetus, which is observed during pregnancy complicated by GDM, some interventions are performed to decrease the risk of GDM by implementing some special diets and supplements. Reducing the prevalence of GDM would improve the health of women and future generations.

It is postulated that in the prevention of GDM, some interventions, such as adherence to a diet as healthy as the MedDiet, optimal physical activity, supplementing vitamin D, fiber, myoinositol, probiotics containing Lactobacillus rhamnosus, and Bifidobacterium lactis could become standard practice. The effectiveness of application of several of the methods mentioned above simultaneously should also be tested, as it could be more effective than applying them individually. Most interventions performed during pregnancy are not entirely successful in GDM prevention, and although there is evidence that some supplements can be beneficial, these trials demonstrate only limited evidence. However, pre-pregnancy dietary patterns appear to reduce the risk of GDM. Programs that introduce appropriate dietary patterns for women before pregnancy appear to be the first line of GDM prevention.

It is necessary to emphasize the difficulty in drawing clear conclusions based on the analyzed publications due to the heterogeneity of the evaluated studies, heterogeneous groups of patients from different geographic regions, and variation in the elements of the interventions, such as duration, intensity and frequency, non-standardized care in the control group, and inconsistent definitions of GDM.

Although several prospective studies have revealed a correlation between some nutrients or dietary patterns and the prevalence of GDM, further studies are needed to evaluate the effects of supplements in large, multicenter, randomized trials involving a wider group of patients, such as non-Caucasian and overweight and obese women.

Implementing an optimal diet could help control glycemia and decrease maternal and fetal complications during pregnancy and postpartum.

## Data Availability

The data used to support the findings of this study are included within the article.
